# Development of a High‐Risk Medication List for Australian Residential Aged Care: A Modified Delphi Study

**DOI:** 10.1111/ajag.70141

**Published:** 2026-02-26

**Authors:** Amanda J. Cross, Madiha Chaudhry, Darshna Goordeen, Juanita L. Breen, Malcolm Clark, Stephanie Daly, Belinda Delardes, Bente Hart, Deborah Hawthorne, Peter J. Hayball, Sarah N. Hilmer, Lisa Kouladjian O’Donnell, MaryAnn Kulh, Kenneth Lee, David F. L. Liew, Stephen Macfarlane, Elizabeth Manias, Anthony Marinucci, Constance Dimity Pond, Helen Rawson, Susan Slatyer, Andrew Stafford, Amy B. Thomson, Kate Wang, Kirolos Wasef, Jonathan Zimmerman, Nadine E. Andrew, Gauri P. Godbole, Louise Lord, Atish Manek, Brigid McInerney, Michelle Steeper, Justin P. Turner, J. Simon Bell

**Affiliations:** ^1^ Centre for Medicine Use and Safety, Faculty of Pharmacy and Pharmaceutical Sciences Monash University Parkville Victoria Australia; ^2^ Wicking Dementia Research and Education Centre University of Tasmania Hobart Tasmania Australia; ^3^ Department of General Practice and Primary Care, Faculty of Medicine, Dentistry and Health Sciences University of Melbourne Parkville Victoria Australia; ^4^ Royal Australian College of General Practitioners East Melbourne Victoria Australia; ^5^ Centre for Research and Evaluation, Ambulance Victoria Blackburn North Victoria Australia; ^6^ Department of Paramedicine Monash University Frankston Victoria Australia; ^7^ Southern New South Wales Local Health District NSW Health Braidwood New South Wales Australia; ^8^ Centre for Optimisation of Medicines, School of Health and Clinical Sciences University of Western Australia Perth Western Australia Australia; ^9^ South Australian Ambulance Service Clinical Services Directorate Eastwood South Australia Australia; ^10^ Faculty of Medicine and Health, Kolling Institute The University of Sydney and Northern Sydney Local Health District St Leonards New South Wales Australia; ^11^ School of Pharmacy, Faculty of Medicine and Health The University of Sydney Camperdown New South Wales Australia; ^12^ School of Medicine and Psychology Australian National University Canberra Australian Capital Territory Australia; ^13^ Department of Clinical Pharmacology and Therapeutics Austin Health Heidelberg Victoria Australia; ^14^ Department of Medicine University of Melbourne Parkville Victoria Australia; ^15^ Department of Psychiatry, Faculty of Medicine, Nursing and Health Sciences Monash University Clayton Victoria Australia; ^16^ Dementia Support Australia Melbourne Victoria Australia; ^17^ Monash Nursing and Midwifery, Faculty of Medicine, Nursing and Health Sciences Monash University Clayton Victoria Australia; ^18^ Royal Australian College of General Practitioners North Sydney New South Wales Australia; ^19^ School of Nursing, College of Health and Education Murdoch University Murdoch Western Australia Australia; ^20^ Curtin School of Diagnostic and Therapeutic Sciences, and enAble Institute, Faculty of Health Sciences Curtin University Perth Western Australia Australia; ^21^ New South Wales Poisons Information Centre Westmead New South Wales Australia; ^22^ Pharmacy, School of Health and Biomedical Sciences RMIT University Bundoora Victoria Australia; ^23^ Goodwin Aged Care Canberra Australian Capital Territory Australia; ^24^ Health of Older Persons Bayside Health (Alfred Care Group) Melbourne Victoria Australia; ^25^ Faculty of Medicine, Nursing and Health Sciences, Peninsula Clinical School Monash University Frankston Victoria Australia; ^26^ Central Coast Local Health District Gosford Hospital Gosford New South Wales Australia; ^27^ Pharmacy Department Royal Melbourne Hospital Parkville Victoria Australia; ^28^ Department of General Practice, School of Public Health and Preventive Medicine Monash University Melbourne Victoria Australia; ^29^ Pharmacy Department Monash Health Clayton Victoria Australia; ^30^ Faculty of Medicine, Dentistry and Health Sciences University of Melbourne Parkville Victoria Australia

**Keywords:** Delphi technique, long‐term care, medication errors, nursing homes

## Abstract

**Introduction:**

High‐risk medications are medications associated with significant patient harm or death if misused or used in error. This study aimed to develop a national consensus high‐risk medication list for use in Australian residential aged care.

**Methods:**

A 3‐round modified Delphi study involving Australian healthcare professionals was conducted. In Round 1, participants indicated their level of agreement, on a 9‐point Likert scale, whether 60 medications/medication classes were considered high‐risk and should be included in a high‐risk medication list for Australian residential aged care. Round 2 included medications/medication classes that did not reach consensus and new medications identified by participants. Consensus was defined as 70% or more of participants responding at 7 or higher on the Likert scale. In Round 3, participants were asked to prioritise medications/medication classes that reached consensus in Round 1 or 2.

**Results:**

In total, 42 participants completed Round 1, and 35 (83%) completed all three rounds. Participants included pharmacists (*n* = 21), prescribers (*n* = 15), nurses (*n* = 5) and a paramedic (*n* = 1), with representation from all Australian states and mainland territories. Overall, 26 medications reached consensus (21 in Round 1, five in Round 2) and were categorised into 15 medications/medication classes for prioritisation in Round 3. The final prioritisation list was opioids, insulin, benzodiazepines, anticoagulants, z‐drugs, antipsychotics, lithium, sulfonylureas with high risk of hypoglycaemia, chemotherapeutic agents, methotrexate, digoxin, narrow therapeutic range antiepileptics, tricyclic antidepressants, immunosuppressants for transplant and sedating antihistamines.

**Discussion:**

This is the first, national consensus list of high‐risk medications developed specifically for Australian residential aged care. It can be used to implement targeted strategies to minimise medication‐related harm.

## Practice Impact

This study developed the first high‐risk medication list specific to Australian residential aged care. The OZ‐ABCD list (Opioids, Z‐drugs and benzodiazepines, Antipsychotics and lithium, Blood thinners, Chemotherapeutic agents and methotrexate, and Diabetes agents with high‐risk of hypoglycaemia) can be used to identify high‐risk medications and support their safe use.

## 1 Introduction

The global cost of medication errors is USD$42 billion per annum [1], with the highest rates of preventable medication‐related harm occurring in older people [2]. Each additional high‐risk medication prescribed to frail older people is associated with a 1.29 times higher risk of repeated hospitalisations based on single variable logistic regression [3]. In the United States of America (USA), anticoagulants and diabetes medications were the most frequent medication types associated with emergency department visits among older adults [4]. Opioids, diabetes medications and anticoagulants were also among the top four most common medications involved in medication errors in North Carolina nursing homes [5]. In Australia, people living in residential aged care (also known as nursing homes or long‐term care facilities) experience a median of three medication‐related adverse events over a 12‐month period, with antithrombotic agents and opioid analgesics among the top five most commonly prescribed classes of medicines prescribed in residents experiencing medication‐related adverse events [6]. Strengthening structures and processes to support safe and effective medication use is key to minimising medication‐related harm [7].

High‐risk medications, sometimes referred to as high‐alert medications, are medications associated with significant patient harm or death if misused or used in error [8, 9]. High‐risk medications are not necessarily inappropriate, although high‐risk medications may be inappropriate for specific individuals and circumstances [9, 10]. The Australian National Safety and Quality Health Service (NSQHS) Standard on Medication Safety requires health services to identify high‐risk medications and take appropriate action to ensure safe storage, prescribing, dispensing and administration [11]. The Australian Government's Strengthened Aged Care Quality Standards (standard 5.3.4) requires aged care providers to establish clear policies, procedures and processes for high‐risk medications [12]. This includes implementing ‘processes for identifying, documenting, monitoring and reviewing the high‐risk medications prescribed to older people in the service’. However, there is currently no Australian consensus list of high‐risk medications specific to residential aged care.

The Australian Commission on Safety and Quality in Healthcare (ACSQHC)'s Antimicrobials, Potassium and other electrolytes, Insulin, Narcotics, Chemotherapy and Heparin, and Systems (APINCHS) high‐risk medication list is the most widely used high‐risk medication list in Australia [9]. The APINCHS list is not exhaustive and is intended for use in hospitals and acute care. The USA's Institute for Safe Medication Practices (ISMP) developed a high‐alert medication list specific for long‐term care settings [13]. However, differences in resident demographics, prescribing patterns and aged care service delivery mean this list may not be directly applicable to Australia. The aim of this study was to develop a national consensus high‐risk medication list specific to the Australian residential aged care setting. It is anticipated that such a list will inform residential aged care high‐risk medication policies and strategies to minimise medication‐related harm.

## 2 Methods

### 2.1 Study Design

A 3‐round modified Delphi consensus method was used as it enabled iterative consensus‐building via asynchronous participation among a geographically diverse sample of healthcare professionals. The Delphi method was modified by using a predefined list of statements for Round 1, online surveys and a multidisciplinary stakeholder panel. Similar Delphi methods have been used to prioritise high‐risk medications in social care [14] and community [15] settings, and have been used to reach consensus on signs and symptoms suggestive of adverse drug events [16, 17]. This study is reported in accordance with the Guideline on Conducting and Reporting Delphi Studies (CREDES) [18].

### 2.2 Recruitment of the Expert Panel

A purposive sampling strategy was employed through professional networks to recruit a heterogenous panel of multidisciplinary healthcare professionals from across Australia. Invitations to participate were sent via email to clinical pharmacologists, general medical practitioners, geriatricians, nurses, nurse practitioners, paramedics, pharmacists and psychiatrists with experience providing care to residents of Australian residential aged care and/or significant practice experience in medication safety for older adults. Potential participants were encouraged to forward the invitation to other experts in their network and subsequent respondents were reviewed by the research team for eligibility and alignment with the sampling frame.

### 2.3 Generating the Statements

The statements for Round 1 were developed based on a systematic scoping review of the literature and a half‐day expert panel meeting.

The scoping review was conducted by searching MEDLINE (Ovid), EMBASE (Ovid), PsycInfo (Ovid), CINAHL, Scopus, Web of Science and the Cochrane Library from inception to 18 July 2023 for studies across the domains of ‘high‐risk medications’ and ‘residential aged care’. Terms synonymous to both domains, including medical subject headings (MeSH), Emtree terms and key words, were searched (File S1). Forward and backward citation tracking of included studies was performed using Web of Science. Grey literature was not searched as this was considered during preparation for the expert panel (see below). Studies were eligible for inclusion if they provided examples or a list of high‐risk medications in a residential aged care setting. High‐risk medications were preliminarily defined as medications that carry an increased risk of significant patient harm or death if misused or used in error, but author definitions for each eligible study were extracted as part of data extraction. Studies reporting criteria for potentially inappropriate medications such as AGS Beers criteria [10], STOPP/START [19], McLeod criteria [20], Laroche list [21] and PRISCUS list [22] were excluded because high‐risk medications are not necessarily inappropriate and vice versa. Studies that only discussed one specific high‐risk medication (e.g., warfarin) were also excluded. Studies conducted in multiple settings were included if specific data were available for residential aged care (e.g., data on high‐risk medications at hospital discharge to residential aged care). Non‐full text articles (e.g., conference abstracts) were only included where there was sufficient information in the abstract to determine the definition and list of high‐risk medications and setting. Title and abstract screening, full text screening and data extraction were performed independently by two investigators (AJC, MC) with disagreements resolved through discussion with a third investigator (DG). Data were extracted into a pre‐piloted table and included the following details: author, year, country, setting, participants, definition of high‐risk medications and list of high‐risk medications in the study.

The scoping review was supplemented with results from a half‐day meeting of an expert panel in Victoria, Australia in July 2022 to identify medications considered high‐risk at the point of hospital transfer from residential aged care. This expert panel was conducted as part of the Medical Research Future Fund Research Data Infrastructure project, *Optimising health information exchange during aged care transfers*. The expert panel was conducted using the nominal group technique and included three pharmacists, two nurses, one general practitioner, one geriatrician and one paramedic. Panellists had experience prescribing, dispensing, administering or monitoring medications for older people in residential aged care or during hospital admissions.

Medications and medication classes identified by the scoping review and expert panel were grouped according to Anatomical Therapeutical Chemical (ATC) Classification codes [23]. One medication class available over‐the‐counter (vitamin A and D, ATC AA1C) was removed due to low perceived risk when it was given at normal doses and the desire to focus on prescription‐only medication. One medication class not used in Australia (glinides, A10BX) was also removed. Medications with inconsistent or similar terminologies (e.g., antiepileptic and anticonvulsant) were merged. The medication/medication class list was reviewed for face‐validity by a panel of four healthcare professionals (general practitioner, nurse and two pharmacists) prior to inclusion in Round 1 of the Delphi Survey.

### 2.4 Delphi Survey Rounds

A three‐round modified Delphi was conducted from January to April 2024 using Qualtrics Insight Platform. Each round was open for 2 weeks, with the subsequent round sent 1 week after the previous round concluded. Reminder emails were sent to non‐responders 1 week, 10 days and 13 days after each round commenced.

### 2.5 Round 1

Participant information collected at the beginning of Round 1 included gender, geographic region, professional role, years of experience in residential aged care or medication safety. Participants were provided with a definition of both high‐risk medications and a high‐risk medication list at the beginning of each round (File S2). All identified medications and medication classes were formulated into statements to consistently remind participants of the intent of the study. The statements were formatted as ‘I believe (insert medication/medication class) is a high‐risk medication and should be included in a high‐risk medication list for Australian residential aged care’. Participants rated their agreement with each statement using a 9‐point Likert scale (where 1 = ‘strongly disagree’ and 9 = ‘strongly agree’). This scale was selected to capture more nuanced levels of responses than shorter or binary response formats and is a common approach used in Delphi studies [17, 24, 25].

Participants were invited to provide free‐text comments to justify their responses, suggest amendments to medications/medication classes (e.g., focus on specific medications within a class) or add additional medication/medication classes for consideration. Criteria for consensus were defined a priori. Statements from Round 1 were advanced to the final prioritisation round (Round 3) if 70% or more of participants responded with 7 or higher on the Likert Scale [26]. Statements receiving ratings of 7 or higher from 30% or fewer of participants were excluded from subsequent rounds.

### 2.6 Round 2

Statements that did not achieve consensus for inclusion or exclusion in Round 1 were retained for further consideration in Round 2: This included statements amended based on participant feedback in Round 1. Participants were provided with the median response for each statement and de‐identified comments representing differing views of participants in Round 1 to promote reflection in Round 2. Deidentified comments were selected by the research team. New medication or medication class statements based on participant free‐text responses from Round 1 were also included. Statements that reached consensus for inclusion in Round 2 (i.e., 70% or more of participants responding with 7 or higher on the Likert Scale) were included in Round 3. All other statements were excluded.

### 2.7 Round 3

Consensus statements from Rounds 1 and 2 were presented to participants in Round 3 for ranking. The research team condensed statements where possible (e.g., if all medications for a medication class reached consensus, then only the medication class was included). Participants were asked to prioritise the top 10 statements (where 1 = highest priority). The number of medications/medication classes participants had to prioritise was defined a priori. The median score across all participants was calculated where the medication/medication class with the lowest median score was identified as the highest priority. The top ranked statements were determined by calculating the median rank for each statement.

### 2.8 Data Analysis

Data analyses were completed using Qualtrics Insights Platform (Provo, UT) and Microsoft Excel (Microsoft Corporation, Irvine, CA). The Round 1 results and open text responses were reviewed by the core research team prior to commencement of Round 2. Prioritisation results were presented as medians and interquartile ranges (IQRs) due to the asymmetric distribution of results, with the mean score used to differentiate medications that had the same median score. Sub‐group analysis was conducted to compare the median (IQR) results of prioritisation in Round 3 between prescribers and non‐prescriber participants, with mean scores used to order medications/medication classes with the same median score.

## 3 Results

### 3.1 Generating the Statements

The scoping review identified 552 records from database searching, and one additional record from citation searching (File S3). After title, abstract and full‐text screening, 10 studies that collectively described eight unique definitions and lists of high‐risk medications used in residential aged care were included and are described in File S4. The eight high‐risk medication lists were used in Australia [27], the United States [28, 29], France [30], Netherlands [31], Spain [32] and the United Kingdom [33], and one was a narrative review not specific to any country [8].

High‐risk medications and medication classes from the eight studies and the medications prioritised by the expert panel (nine sources in total) are presented in File S5. Nervous system medications were identified in all sources, with analgesic (N02, *n* = 8/9) and psycholeptic (N05, *n* = 8/9) pharmacological subgroups most prevalent. Antithrombotic agents (B01, *n* = 8/9) and drugs used in diabetes (A10, *n* = 7/9) were also common. Sixty medications or medication classes derived from the scoping review and expert panel were used for the Round 1 Delphi.

### 3.2 Delphi Participants

**FIGURE 1:**
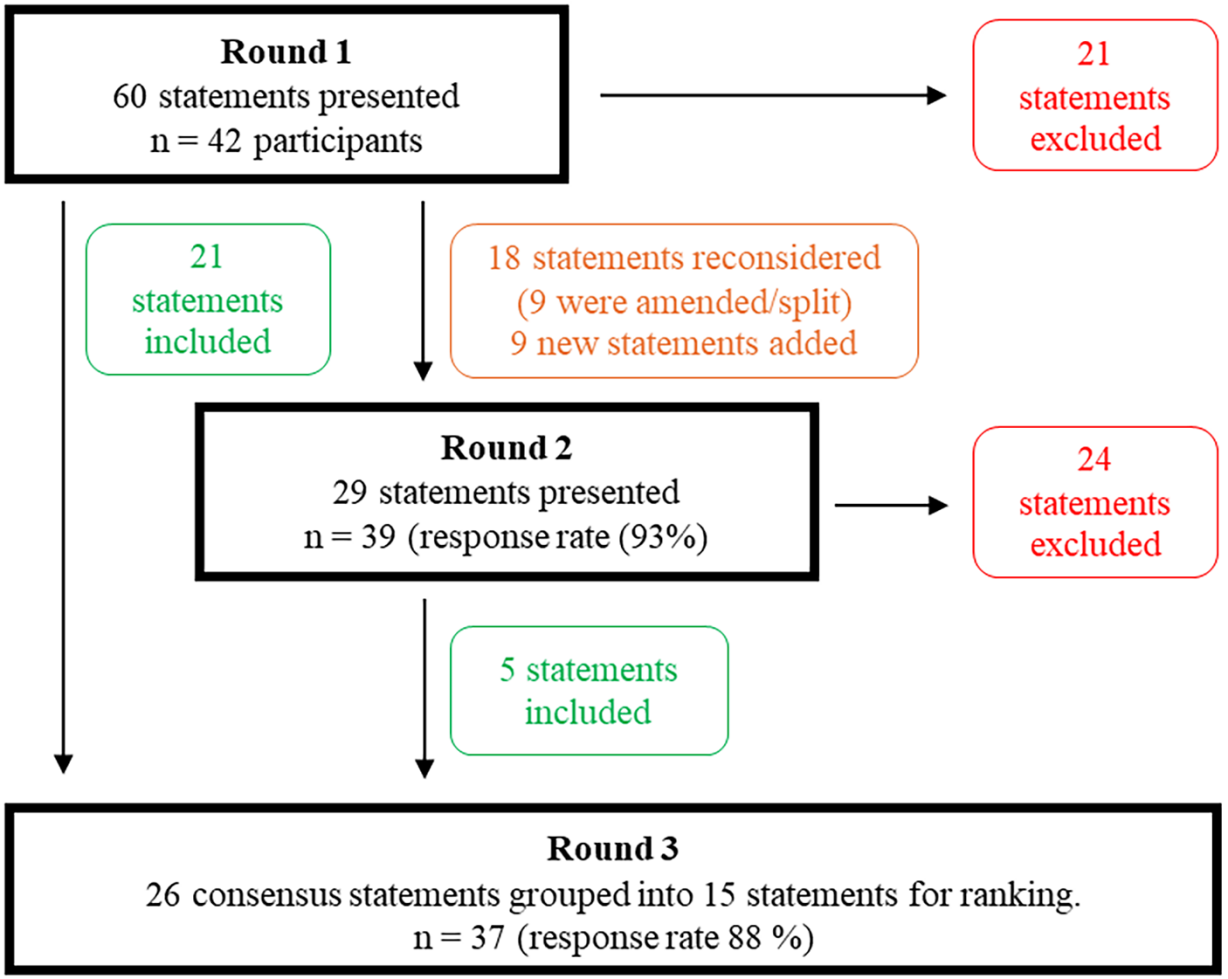
Flow of participants and statements through 3‐round modified Delphi study.

Thirty‐five of 42 (83%) participants completed all three rounds (Figure 1). Participants included pharmacists (*n* = 21), prescribers (*n* = 15, including geriatricians, psychiatrists, general practitioners, a nurse practitioner and a pharmacologist), nurses (*n* = 5) and a paramedic (*n* = 1). Participants were from all states and mainland territories of Australia, with most (*n* = 25, 60%) having 10 or more years' experience in residential aged care or aged care medication safety (Table 1).

**TABLE 1:** Characteristics of Round 1 participants.

Characteristic	No. of participants, *n* (%)
Gender, female	25 (60)
Role	
Pharmacist	21 (50)
Prescriber^a^	15 (36)
Nurse	5 (12)
Paramedic	1 (2)
Number of years of experience providing care in the residential aged care setting or in aged care medication safety	
0–5 years	8 (19)
6–10 years	9 (21)
11–15 years	5 (12)
16–20 years	8 (19)
> 20 years	12 (29)
State/Territory	
Victoria	13 (31)
New South Wales	11 (26)
Western Australia	5 (12)
Queensland	4 (10)
South Australia	3 (7)
Australian Capital Territory	2 (5)
Northern Territory	2 (5)
Tasmania	2 (5)
Geographic region^b^	
Metropolitan	32 (76)
Regional	11 (26)
Rural and/or remote	9 (21)

^a^ Prescribers included general practitioners, geriatricians, psychiatrists, a nurse practitioner and a clinical pharmacologist.

^b^ Not mutually exclusive.

### 3.3 Round 1

Of the 60 statements presented to participants in Round 1, 21 (35%) reached consensus for inclusion (Figure 1). The level of agreement ranged from 74% for ‘codeine’ and ‘all oral anticoagulants’, to 98% for ‘methadone’ (Table 2). Twenty‐one statements (35%) reached consensus for exclusion. Of the 18 statements that did not reach consensus and were included in Round 2, half (9/18) were amended based on participant suggestions. A further nine statements were also added for consideration in Round 2.

**TABLE 2:** Delphi results—Rounds 1, 2 and 3.

Round 1 statement: ‘I believe (insert medication/medication class) is a high‐risk medication and should be included on a high‐risk medication list for Australian residential aged care’		Proportion of participants who rated ≥ 7; *n* (%)	Round 2 statements: ‘I believe (insert medication/medication class) is a high‐risk medication and should be included on a high‐risk medication list for Australian residential aged care’	Proportion of participants who rated ≥ 7; *n* (%)	Round 3	Median prioritisation (where 1 = highest priority)
Category 1—Alimentary tract and metabolism	Insulin	37 (88)	N/A	N/A	Insulin	4
	Metformin	2 (5)	N/A	N/A	N/A	N/A
	All sulfonylureas	25 (60)	Sulfonylureas with low/intermediate risk of hypoglycaemia	14 (36)	N/A	
			Sulfonylureas with high risk of hypoglycaemia	24 (87)	Sulfonylureas with high‐risk of hypoglycaemia	7
			All sulfonylureas	17 (44)		
	All oral hypoglycemic medications	7 (17)	N/A	N/A	N/A	N/A
	All anti‐diabetic agents	7 (17)	N/A	N/A	N/A	N/A
	Vitamin D and vitamin D analogues	2 (5)	Calcitriol	4 (10)	N/A	N/A
	Calcium	2 (5)	N/A	N/A	N/A	N/A
Category 2—Blood and blood forming organs	Warfarin	38 (91)	N/A	N/A	Warfarin	N/A^a^
	All oral anticoagulants	31 (74)	N/A	N/A	All oral anticoagulants	N/A^a^
	All anticoagulants	32 (76)	N/A	N/A	All anticoagulants	6
	All antiplatelets (including aspirin)	19 (45)	All antiplatelets (including aspirin)	18 (46)	N/A	N/A
	Iron dextran (parenteral)	14 (33)	All parenteral iron	6 (15)	N/A	N/A
	All parenteral nutritional preparations	14 (33)	All parenteral nutritional preparations	11 (28)	N/A	N/A
	All potassium and other electrolytes	23 (55)	Potassium (all dosage forms)	27 (69)	N/A	N/A
Category 3—Cardiovascular system	Propranolol	20 (48)	Propranolol	19 (49)	N/A	N/A
	All beta‐blockers	17 (41)	All beta‐blockers	10 (26)	N/A	N/A
	All antihypertensive agents	9 (21)	N/A	N/A	N/A	N/A
	Digoxin	32 (76)	N/A	N/A	Digoxin	9
	Adrenaline (parenteral)	28 (67)	Adrenaline	26 (67)	N/A	N/A
	Eplerenone	12 (29)	N/A	N/A	N/A	N/A
	Spironolactone	11 (26)	N/A	N/A	N/A	N/A
	All loop diuretics	13 (31)	All loop diuretics	8 (21)	N/A	N/A
	All diuretics	9 (21)	N/A	N/A	N/A	N/A
			Non‐dihydropyridine calcium channel blockers	14 (36)	N/A	N/A
			Amiodarone	26 (66.7)	N/A	N/A
Category 4—Musculo‐skeletal system	All bisphosphonates	6 (14)	N/A	N/A	N/A	N/A
	Drugs other than bisphosphonates that affect bone structure and mineralisation	5 (12)	N/A	N/A	N/A	N/A
	All topical products for joint and muscular pain	11 (26)	N/A	N/A	N/A	N/A
	All NSAIDs	19 (45)	All NSAIDs	21 (54)	N/A	N/A
	All anti‐inflammatory medications and anti‐rheumatic medications	18 (43)	All anti‐rheumatic medications	15 (39)	N/A	N/A
Category 5—Nervous system	Paracetamol	4 (10)	N/A	N/A	N/A	N/A
	Morphine	39 (93)	N/A	N/A	Morphine	N/A^a^
	Methadone	41 (98)	N/A	N/A	Methadone	N/A^a^
	Codeine	31 (74)	N/A	N/A	Codeine	N/A^a^
	Oxycodone	39 (93)	N/A	N/A	Oxycodone	N/A^a^
	Tramadol	38 (91)	N/A	N/A	Tramadol	N/A^a^
	All opioids	40 (95)	N/A	N/A	All opioids	3
	All analgesics	8 (19)	N/A	N/A	N/A	N/A
	Carbamazepine	24 (57)	Carbamazepine	25 (64)	N/A	N/A
	Narrow therapeutic range antiepileptics	28 (67)	Narrow therapeutic range antiepileptics	32 (82)	Narrow therapeutic range antiepileptics	9
	All antiepileptics	20 (48)	All antiepileptics	16 (41)	N/A	N/A
	All anti‐Parkinson medications	18 (43)	All anti‐Parkinson medications	16 (41)	N/A	N/A
	Amitriptyline	23 (55)	Tricyclic antidepressants (e.g., amitriptyline)	31 (80)	Tricyclic antidepressants	12
	Mirtazapine	9 (21)	N/A	N/A	N/A	N/A
	Sertraline	7 (17)	N/A	N/A	N/A	N/A
	All antidepressants	8 (19)	N/A	N/A	N/A	N/A
	Lithium	37 (88)	N/A	N/A	Lithium	7
	Haloperidol	36 (86)	N/A	N/A	Haloperidol	N/A^a^
	Quetiapine	34 (81)	N/A	N/A	Quetiapine	N/A^a^
	All antipsychotics	34 (81)	N/A	N/A	All antipsychotics	7
	Diazepam	37 (88)	N/A	N/A	Diazepam	N/A^a^
	All benzodiazepines	37 (88)	N/A	N/A	All benzodiazepines	5
	All z‐drugs	38 (91)	N/A	N/A	All z‐drugs	7
			Gabapentinoids (e.g., pregabalin, gabapentin)	27 (69)	N/A	N/A
			Medications for dementia (e.g., donepezil, memantine)	10 (26)	N/A	N/A
Category 6—Systemic hormonal preparations	Corticosteroids for long term use	20 (48)	Oral corticosteroids	19 (49)	N/A	N/A
	Calcitonin	11 (26)	N/A	N/A	N/A	N/A
	All parathyroid hormones	10 (24)	N/A	N/A	N/A	N/A
Category 7—Anti‐infectives	All systemic antibiotics	13 (31)	All systemic antibiotics (e.g., oral and IV)	5 (13)	N/A	N/A
	All antimicrobials	8 (19)	N/A	N/A	N/A	N/A
Category 8—Antineoplastic and immunomodulating agents	All oral cytostatics	36 (86)	N/A	N/A	All oral cytostatics	N/A^a^
	All chemotherapeutic agents	36 (86)	N/A	N/A	All chemotherapeutic agents	9
	Methotrexate	35 (83)	N/A	N/A	Methotrexate	9
			All immunosuppressant medications for transplant	29 (74)	All immunosuppressant medications for transplant	14
	Selective oestrogen receptor modulators	11 (26)	N/A	N/A	N/A	N/A
Category 9—(new medications)			Urinary anticholinergics (oxybutynin)	26 (67%)	N/A	N/A
			All drugs for urinary frequency and incontinence	16 (41%)	N/A	N/A
			Sedating antihistamines	28 (72%)	Sedating antihistamines	15

^a^ Medication was collapsed under its broader medication class for Round 3.

### 3.4 Round 2

Of the 29 Round 2 statements, 5 (17%) reached consensus for inclusion and 24 (83%) were excluded (Figure 1).

### 3.5 Round 3

The 26 statements that reached consensus for inclusion in Rounds 1 or 2 were grouped into 15 final medications or medication classes and ranked by participants (Table 2). The top eight ranked medications overall were opioids, insulin, benzodiazepines, anticoagulants, z‐drugs, lithium, sulfonylureas with high risk of hypoglycaemia and antipsychotics (Table 3). Chemotherapeutic agents, methotrexate, digoxin and narrow therapeutic range antiepileptics were the next top ranked medications, all with a median score of 9, but when ordered based on mean score chemotherapeutic agents and methotrexate were prioritised higher by participants. The mnemonic OZ‐ABCD was developed through consultation with the multidisciplinary co‐author team to assist end‐users in identifying the top 10 high‐risk medications. The additional five medications or medication classes that reached consensus but were not rated in the top 10 by the participants are included below the mnemonic for completeness (Box 1).

**TABLE 3:** Median (IQR) scores for Round 3 for all participants, and prescribers vs. nurses and pharmacists.

Medication or medication class	Median (IQR)	Median (IQR) for prescribers^a^ (*n* = 14)	Median (IQR) for nurses and pharmacists (*n* = 23)
Opioids	3 (1–5)	3.5 (3–4.75)	2 (1–5.5)
Insulin	4 (2–5)	2.5 (2–5)	4 (2–5)
Benzodiazepines	5 (3–9)	4 (3–6)	7 (3–11)
Anticoagulants	6 (3–10)	6 (3.25–9.5)	6 (3.5–9.5)
Z‐drugs	7 (4–11)	5 (2.25–7.5)	9 (4.5–11.5), mean 8.4
Lithium	7 (6–10)	9 (7.25–10.75)	6 (4.5–10)
Sulfonylureas with high‐risk of hypoglycaemia	7 (6–10)	6 (5.25–9.5)	7 (6–11)
Antipsychotics	7 (4–10)	5.5 (4–9.75)	9 (5–10.5), mean 7.8
Chemotherapeutic agents	9 (3–11), mean 7.7	8.5 (3.75–10.75)	9 (3–11.5), mean 8.0
Methotrexate	9 (5–12), mean 8.4	10.5 (9.25–12), mean 10.1	8 (3.5–11)
Digoxin	9 (5–14), mean 9.3	14 (11.25–14.75)	7 (4.5–12)
Narrow therapeutic range antiepileptics	9 (7–12), mean 9.5	12 (8.25–13)	9 (7–10), mean 8.6
Tricyclic antidepressants	12 (9–13)	11.5 (8.25–13)	12 (9.5–13)
Immunosuppressant medications for transplant	14 (8–14)	10.5 (7–14), mean 10.3	14 (9–15)
Sedating antihistamines	15 (10–15)	15 (11.25–15)	15 (8–15)

*Note:* Shaded cells indicate top 10 ranked medications for each group. Mean scores were presented when two or more medications had same median score. Lowest mean score was used to order medications/medication classes with same median score.

Abbreviation: IQR, interquartile range.

^a^ Prescribers include geriatricians, psychiatrists, general practitioners, nurse practitioners, and pharmacologists.

BOX 1 **OZ‐ABCD high‐risk medication list for Australian residential aged care.**

**Table:** 

O	Opioids
Z	Z‐drugs and benzodiazepines
A	Antipsychotics and lithium
B	Blood thinners (anticoagulants)
C	Chemotherapeutic (anti‐cancer) agents and methotrexate
D	Diabetes agents with high‐risk of hypoglycaemia (insulin, sulfonylurea)

Other high‐risk medications: digoxin, narrow therapeutic range antiepileptics, tricyclic antidepressants, immunosuppressants for transplant, and sedating antihistamines.

The top 10 prioritised medications or medication classes overall were the same as the top 10 for the prescriber participant subgroup scores. The nursing and allied health subgroup prioritised digoxin in their top 10, instead of Z‐drugs (Table 3).

## 4 Discussion

The 15 medications and medication classes prioritised by the multidisciplinary panel in this study represent the first national consensus high‐risk medication list for Australian residential aged care. The OZ‐ABCD list provides a concise and practical tool to support risk mitigation and avoid medication‐related harm.

The OZ‐ABCD list addresses a clear gap in medication safety tools for Australian residential aged care. When comparing to other established high‐risk medication lists, opioids, insulin, anticoagulants and chemotherapeutic agents are consistently identified, suggesting their risk is agnostic of practice setting [9, 13, 15]. However, there were also key differences in the OZ‐ABCD list when comparing to acute high‐risk medication lists, such as the omission of anti‐infectives and potassium. While these medications likely still represent risk, their lower frequency of use and potential for harm may have influenced the panel when prioritising medications for a residential aged care specific high‐risk medication list. The inclusion of hypnotics (benzodiazepines and z‐drugs) in the OZ‐ABCD list is a key difference when comparing to the ISMP high‐alert medication list for long‐term care [8]. Inclusion of hypnotics is consistent with hypnotics being the second most implicated medication class associated with errors in residential aged care [5]. The OZ‐ABCD list is distinct from all current lists used in residential aged care as identified in the literature review as part of this study. Dumitrescu et al.'s high‐risk medication list for home care nursing, developed by experts from four European countries [15], includes nine of the top 10 medications in the OZ‐ABCD list, likely reflecting the similar demographics of people accessing home care nursing and residential aged care. However Dumitrescu's list includes 27 high‐risk medications and medication classes meaning it may be less practical for prospective use by clinicians at the point‐of‐care. Some of the medications included in Dumitrescu et al.'s list were not prioritised by our panel and were thus excluded from our final list (e.g., anti‐arrhythmics and carbamazepine). This underscores the importance of setting and country specific high‐risk medication lists, further highlighting the gap that the OZ‐ABCD list seeks to fill.

The OZ‐ABCD list provides a foundation to identify, monitor and review high‐risk medication use, which is consistent with Australia's new aged care standards [12] and the Australian Government's response to the WHO Global patient safety challenge: Medication without harm [34]. Managing the risks associated with these 15 medications will likely require different strategies, tailored to the specific risks and potential for harm for each medication in the residential aged care setting. For example, anticoagulants are included due to bleeding risk, which is high‐risk for residents with specific comorbidities and heightened susceptibility to falls and fall‐related injuries [35].

Chemotherapeutic agents may not always be clearly labelled or stored securely in residential aged care settings, and are high‐risk due to their narrow therapeutic index, complex dosing requirements and potential for adverse events with even minor dose errors. For example, in residential aged care settings, where residents have a high prevalence of dysphagia, crushing chemotherapy medications that should remain intact could expose residents to hazardous particles [36]. Low‐dose methotrexate for autoimmune non‐oncological indications was also prioritised by panellists, separate to chemotherapy medications, as it has well‐described toxicity from mis‐dosing [37], even if handling risks remain overstated in contrast to chemotherapeutics [38]. The presence of narrow therapeutic index medications or medications that require close monitoring, such as lithium, digoxin and insulin, is consistent across care settings, but may require different strategies to mitigate risk in residential aged care where medication administration and monitoring is often carried out by staff with different levels of training to those who perform these activities in a hospital environment.

The identification of medication classes such as opioids, benzodiazepines, Z‐drugs and antipsychotics as high‐risk medications likely reflects both their high prevalence and propensity to cause harm. With opioids prescribed to up to half of all residents [39, 40], benzodiazepines to a third of residents [41] and antipsychotics to 42% of residents [42], these three medication classes pose a significant and persistent risk. Older adults are more susceptible to adverse effects from central nervous system medications [43, 44], and analgesics and hypnotics are the two most common medication classes involved in medication errors in residential aged care [5]. However, NSAIDs are used less frequently in Australian residential aged care (2% of residents) compared to other countries such as Japan; [45] thus, despite their known risks in this population, this may be why NSAIDs were not prioritised by participants for inclusion in the final list. This is consistent with Dumitrescu et al.'s high‐risk medication list for home care nursing [15]. Resident‐level interventions, such as deprescribing, psychotropic adverse event monitoring [17] and optimisation of non‐pharmacological strategies [45], as well as system‐level interventions, such as enhanced local safety protocols and procedures, targeted healthcare professional training [46] and artificial intelligence supported clinical decision support systems [47], should be considered together to reduce harm and enhance safe use of medication in this setting [48].

Effective implementation of high‐risk medication protocols requires a thorough understanding of the risks and the specific mechanisms through which harm can occur, as well as the frequency of use of each medication in the aged care setting. Onsite pharmacists may be well‐placed to lead implementation of the high‐risk medication list in residential aged care. By acting as knowledge brokers [49, 50], pharmacists can help to identify and communicate the rationale behind each medication's inclusion, support development of context‐specific targeted safety measures, such as clear labelling, prompts in electronic national resident medication charts, modified administration procedures and enhanced staff training and ensure key stakeholders are aware of residents who are taking high‐risk medications (e.g., at transitions of care), ultimately minimising the likelihood of medication‐related harm.

Strengths of this study include the diverse range of participants from all states and mainland territories of Australia, the multidisciplinary participant cohort representing key stakeholders who support medication safety in Australian residential aged care, and the high retention rate between rounds. The online format of the survey facilitated participation across geographical areas and time zones and likely contributed to the high retention rate. Participant responses to the Delphi survey remained confidential and were not shared in an identifiable manner between participants. This quasi‐anonymous approach is a key strength of the Delphi method, as it minimises bias and encourages independent input. Limitations of this study include that the list of high‐risk medications included in Round 1 was developed based on included studies in the literature review, which had different definitions of high‐risk medications. Participants were provided with the definition of high‐risk medications at the start of each round of the Delphi; however, it is possible that panel members approached this definition in different ways, which may have impacted the acuity of the definition of high‐risk medications. As with any Delphi study, the composition and size of the expert panel can influence outcomes, meaning different panels may produce varying results. It is possible that a larger sample size may have increased the replicability of the results; [51] however, the sample size is consistent with or larger than similar recent Delphi studies [15, 16, 17]. Sub‐group analysis comparing prescribers to non‐prescribers was conducted given the difference in scope of practice, responsibilities and clinical decision‐making authority between these two groups; however, interpretation is limited by the small sample size. The broad representation and level of experience across the panel mitigates the chance of major variation. The OZ‐ACBD list is a concise tool developed through consensus opinion, and it has not yet been tested for practicability.

## 5 Conclusions

This study developed the first national consensus list of medications and medication classes considered high‐risk for Australian residential aged care. The OZ‐ABCD list provides healthcare professionals and aged care providers with a practical tool to help identify, document, monitor and review high‐risk medication use, and the associated potentially avoidable medication‐related harm. The list provides a foundation for risk mitigation strategies, and future research and practice should focus on strategies to support uptake and implementation of the list in residential aged care settings.

## Funding

A.J.C. is supported by an NHMRC Emerging Leadership 1 grant (APP2009633). A component of this research was also funded by a Medical Research Future Fund (MRFF) Primary Care Data Infrastructure Grant (PHRDI000008). K.L. is supported by the Western Australian Future Health Research and Innovation Fund/Western Australian Department of Health, Grant ID WANMA/EL2023‐24 Lee.

## Ethics Statement

This study was approved by the Monash University Human Research Ethics Committee (ID 40244). The expert panel was also approved by the Monash University Human Research Ethics Committee (ID 32880).

## Conflicts of Interest

A.J.C. has received grant or consulting funds from the National Health and Medical Research Council, Medical Research Future Fund, Dementia Australia Research Foundation and the Pharmaceutical Society of Australia. All these funds were paid to the administering University. A.J.C. is also a national board director for the Pharmaceutical Society of Australia. S.D. has received paid Honoraria from Roche, Biogen, Lily, Eisai and holds a contract with Medicines Australia for the Brain Health Collective. S.D. is also the owner of Sensus Cognition, a primary care cognition clinic. C.D.P. has received grant or consulting funds from the National Health and Medical Research Council, Medical Research Future Fund, Dementia Training Australia, Commonwealth Department of Health and Aged Care, Medicines Australia, Roche Pharmaceuticals and Biogen. A.S. has received consulting funds to provide medication management services to residential aged care homes. He has also received grant funds from the National Health and Medical Research Council, Medical Research Future Fund, Dementia Centre for Research Collaboration and the Pharmaceutical Society of Australia. All these funds were paid to the administering University. A.M. is an owner and director of Aged Care GP. J.S.B. has received grant or consulting funds from the NHMRC, Medical Research Future Fund, Victorian Government Department of Health and Human Services, Dementia Australia Research Foundation, Yulgilbar Foundation, Aged Care Quality and Safety Commission, Australian Commission on Safety and Quality in Health Care, Dementia Centre for Research Collaboration, Pharmaceutical Society of Australia, Advanced Pharmacy Australia (formerly Society of Hospital Pharmacists of Australia), GlaxoSmithKline Supported Studies Programme, Amgen, and several aged care provider organisations. All these funds were paid to the administering University. K.W. is an associate editor of the Australasian Journal on Ageing. All other authors declare they have no conflicts of interest relevant to this work.

## Supporting information


**File S1:** ajag70141‐sup‐0001‐FileS1.docx.

**File S2:** ajag70141‐sup‐0002‐FileS2.docx.

**File S3:** ajag70141‐sup‐0003‐FileS3.docx.

**File S4:** ajag70141‐sup‐0004‐FileS4.docx.

**File S5:** ajag70141‐sup‐0005‐FileS5.docx.

**Appendix S1:** ajag70141‐sup‐0006‐AppendixS1.docx.

## Data Availability

The data that support the findings of this study are available upon request from the corresponding author. The data are not publicly available due to privacy or ethical restrictions.
