# Supplementary material for: Development of a High‐Risk Medication List for Australian Residential Aged Care: A Modified Delphi Study
Source: Australas J Ageing. 2026 Feb 26;45(1):e70141. doi: 10.1111/ajag.70141 (PMC12945874; doi:10.1111/ajag.70141)
Supplement: Supplementary file 1 — File S1: ajag70141‐sup‐0001‐FileS1.docx. [file AJAG-45-0-s006.docx]

**Supplementary File 1:** Search strategy for systematic scoping review

**All searches were run on July 18^th^ 2023**

**Medline (Ovid)**

| **Line** | **Search** | **Hits** |
| --- | --- | --- |
| 1 | (high-risk or high-alert or high-hazard).mp. | 315832 |
| 2 | (medicine* or medication* or drug* or pharmaceutic* or therapeutic*).mp. | 8444407 |
| 3 | (nursing home* or long-term care or residential care or residential facilit* or veteran home* or care home* or old age home* or nursing facilit* or intermediate care or aged care).mp. or exp residential facilities/ or exp long-term care/ | 113822 |
| 4 | (1 adj2 2) AND 3 | 56 |

**EMBASE (Ovid)**

| **Line** | **Search** | **Hits** |
| --- | --- | --- |
| 1 | (high-risk or high-alert or high-hazard).mp. | 780257 |
| 2 | (medicine* or medication* or drug* or pharmaceutic* or therapeutic*).mp. | 15995562 |
| 3 | (nursing home* or long-term care or residential care or residential facilit* or veteran home* or care home* or old age home* or nursing facilit* or intermediate care or aged care).mp. or exp residential home/ or exp nursing home/ or exp long term care/ | 2465763 |
| 4 | (1 adj2 2) AND 3 | 468 |

**PsycInfo (Ovid)**

| **Line** | **Search** | **Hits** |
| --- | --- | --- |
| 1 | (high-risk or high-alert or high-hazard).mp. | 47892 |
| 2 | (medicine* or medication* or drug* or pharmaceutic* or therapeutic*).mp. | 717294 |
| 3 | (nursing home* or long-term care or residential care or residential facilit* or veteran home* or care home* or old age home* or nursing facilit* or intermediate care or aged care).mp. or exp nursing homes/ or assisted living/ or residential care institutions/ or long term care/ | 42052 |
| 4 | (1 adj2 2) AND 3 | 11 |

**CINAHL**

| **Line** | **Search** | **Hits** |
| --- | --- | --- |
| S1 | “high risk” or “high alert” or “high hazard” | 109198 |
| S2 | medicine* or medication* or drug* or pharmaceutic* or therapeutic* | 1673912 |
| S3 | “nursing home∗” OR “long term care” OR “residential care” OR “residential facility*” OR “veteran home*” OR “care home*” OR “old age home∗” OR “nursing facility*” OR “intermediate care” OR “aged care” | 77388 |
|  | MH “Nursing Homes+” OR “Long Term Care” | 59549 |
| S4 | (S1 N2 S2) AND (S3 OR S4) | 46 |

**Scopus**

| **Search** | **Hits** |
| --- | --- |
| ( TITLE-ABS-KEY ( "high-risk" OR "high-alert" OR "high-hazard" ) ) | 647133 |
| ( TITLE-ABS-KEY ( medicine* OR medication* OR drug* OR pharmaceutic* OR therapeutic* ) ) | 12298027 |
| ( TITLE-ABS-KEY ( "nursing home*" OR "long-term care" OR "residential care" OR "residential facilit*" OR "veteran home*" OR "care home*" OR "old age home*" OR "nursing facilit*" OR "intermediate care" OR "aged care" ) ) | 248906 |
| ( ( TITLE-ABS-KEY ( "high-risk" OR "high-alert" OR "high-hazard" ) ) W/2 ( TITLE-ABS-KEY ( medicine* OR medication* OR drug* OR pharmaceutic* OR therapeutic* ) ) ) AND ( TITLE-ABS-KEY ( "nursing home*" OR "long-term care" OR "residential care" OR "residential facilit*" OR "veteran home*" OR "care home*" OR "old age home*" OR "nursing facilit*" OR "intermediate care" OR "aged care" ) ) | 122 |

**Web of Science**

| **Search** | **Hits** |
| --- | --- |
| "high-risk" OR "high-alert" OR "high-hazard" | 407022 |
| medicine* OR medication* OR drug* OR pharmaceutic* OR therapeutic* | 7795500 |
| "nursing home*" OR "long-term care" OR "residential care" OR "residential facilit*" OR "veteran home*" OR "care home*" OR "old age home*" OR "nursing facilit*" OR "intermediate care" OR "aged care" | 103997 |
| (("high-risk" OR "high-alert" OR "high-hazard") NEAR/2 (medicine* OR medication* OR drug* OR pharmaceutic* OR therapeutic*)) AND ("nursing home*" OR "long-term care" OR "residential care" OR "residential facilit*" OR "veteran home*" OR "care home*" OR "old age home*" OR "nursing facilit*" OR "intermediate care" OR "aged care") | 86 |

**Cochrane**

| **Search** | **Hits** |
| --- | --- |
| ("high-risk" or "high-alert" or "high-hazard") NEXT/2 (medicine* or medication* or drug* or pharmaceutic* or therapeutic*) in Title Abstract Keyword AND ("nursing home" or "nursing homes" or "long-term care" or "residential care" or "residential facility" or "residential facilities" or "veteran home" or "veteran homes" or "care home" or "care homes" or "old age home" or "old age homes" or "nursing facility" or "nursing facilities" or "intermediate care" or "aged care") in Title Abstract Keyword | 20 |

Total = 809 (before deduplication) as of July 18th, 2023
