# Supplementary material for: Development of a High‐Risk Medication List for Australian Residential Aged Care: A Modified Delphi Study
Source: Australas J Ageing. 2026 Feb 26;45(1):e70141. doi: 10.1111/ajag.70141 (PMC12945874; doi:10.1111/ajag.70141)
Supplement: Supplementary file 2 — File S2: ajag70141‐sup‐0002‐FileS2.docx. [file AJAG-45-0-s001.docx]

Supplementary File 2: Round 1 Survey

**Round 1 Survey Questions**

******* Page 1 *******

**Project ID**: 40244

**Project title**: Development of a high-risk medication list for Australian Residential Aged Care: A Delphi Study

**Chief investigator**: Dr Amanda Cross

I have been invited to take part in the Monash University research project specified above. I have read and understood the Explanatory Statement and I hereby consent to participate in the project.

- Yes
- No

******* Page 2 *******

Please enter the following to allow us to describe the overall characteristics of participants in this study

1. Gender ___________
2. State or Territory where you work majority of the time (select one)
   - ACT
   - NSW
   - NT
   - Qld
   - SA
   - Tas
   - Vic
   - WA
3. Geographic region where you work (select all that apply)
   1. Metropolitan
   2. Regional
   3. Rural
   4. Remote
4. Role (e.g. general practitioner, pharmacist, nurse) _______________
5. Number of years providing care for older adults in the residential aged care setting or number of years of relevant practice experience in quality use of medications for older adults ____________

******* Page 3 *******

Participant instructions

You will be presented with medications or medication classes that have been identified in the literature as high-risk medications in residential aged care.

In the case of medication classes, some examples of common medications have been provided to aid understanding, but the examples are not intended to be a comprehensive list of all medications in that medication class.

Medications/medication classes will be presented in 8 categories (according to the World Health Organisation Anatomical Therapeutical Chemical [ATC] Classification system). Sub-categories based on the pharmacological sub-groups have been provided for some categories.

**For each category/sub-category, we would like you to:**

1. **Rate your level of agreement on whether these medications and/or medication classes are high-risk medications AND should be included in a high-risk medication list for use in Australian residential aged care.**
2. Provide a brief written reason for your scoring (*optional*).
3. Suggest any changes to the terminology used to describe the medication/medication classes presented or suggest additional medication/medication classes in the category you feel are important (*optional*).

For your reference, we ask you to consider the following information:

A **high-risk medication** is defined as a medication that increases the risk of significant harm or death if it is used in error (e.g. wrong drug, wrong dose, wrong route, wrong resident).

A **high-risk medication list** is a list of high-risk medications relevant to the setting. The National Safety and Quality Health Service (NSQHS) Standard on Medication Safety requires health services to identify high-risk medications used locally within their organisation and take appropriate action to ensure that they are stored, prescribed, dispensed and administered safely.

High-risk medications are defined differently to potentially inappropriate medications. Potentially inappropriate medications are defined as medications that should be avoided due to the high risk of adverse events in this population and/or insufficient evidence of benefits. It is possible that some medications may be considered both a high-risk medication and a potentially inappropriate medication.

******* Page 4 *******

**Category 1 – Alimentary Tract and Metabolism**

***The following medications and/or medication classes relate to medications used in diabetes.***

I believe INSULIN is a high-risk medication and should be included in a high-risk medication list for use in Australian residential aged care

Strongly disagree 1 2 3 4 5 6 7 8 9 Strongly agree

I believe METFORMIN is a high-risk medication and should be included in a high-risk medication list for use in Australian residential aged care

Strongly disagree 1 2 3 4 5 6 7 8 9 Strongly agree

I believe ALL SULPHONYLUREAS *(e.g. glibenclamide, gliclazide, glimepiride)* should be considered high-risk medications and should be included in a high-risk medication list for use in Australian residential aged care

Strongly disagree 1 2 3 4 5 6 7 8 9 Strongly agree

I believe ALL ORAL HYPOGLYCEMIC MEDICATIONS should be considered high-risk medications and should be included in a high-risk medication list for use in Australian residential aged care

Strongly disagree 1 2 3 4 5 6 7 8 9 Strongly agree

I believe ALL ANTI-DIABETIC AGENTS should be considered high-risk medications and should be included in a high-risk medication list for use in Australian residential aged care

Strongly disagree 1 2 3 4 5 6 7 8 9 Strongly agree

Comments to justify responses (optional) _____________________________

Suggested changes in terminology or additional medication/medication classes for this category (optional) _____________________

***The following medications and/or medication classes relate to vitamins and mineral supplements.***

I believe VITAMIN D and VITAMIN D ANALOGUES *(e.g. calcitriol)* are high-risk medications and should be included in a high-risk medication list for use in Australian residential aged care

Strongly disagree 1 2 3 4 5 6 7 8 9 Strongly agree

I believe Calcium is a high-risk medication and should be included in a high-risk medication list for use in Australian residential aged care

Strongly disagree 1 2 3 4 5 6 7 8 9 Strongly agree

Comments to justify response (optional) _____________________________

Suggested changes in terminology or additional medication/medication classes for this category (optional) _____________________******* Page 5 *******

**Category 2 – Blood and Blood Forming Organs**

***The following medications and/or medication classes relate to antithrombotic agents.***

I believe WARFARIN is a high-risk medication and should be included in a high-risk medication list for use in Australian residential aged care

Strongly disagree 1 2 3 4 5 6 7 8 9 Strongly agree

I believe ALL **ORAL** ANTICOAGULANTS *(e.g. apixaban, rivaroxaban, warfarin)* should be considered high-risk medications and should be included in a high-risk medication list for use in Australian residential aged care

Strongly disagree 1 2 3 4 5 6 7 8 9 Strongly agree

I believe ALL ANTICOAGULANTS *(e.g. apixaban, enoxaparin, heparin, rivaroxaban, warfarin)* should be considered high-risk medications and should be included in a high-risk medication list for use in Australian residential aged care

Strongly disagree 1 2 3 4 5 6 7 8 9 Strongly agree

I believe ALL ANTIPLATELETS (INCLUDING ASPIRIN) *(e.g. clopidogrel, prasugrel, ticagrelor, dabigatran)* should be considered high-risk medications and should be included in a high-risk medication list for use in Australian residential aged care

Strongly disagree 1 2 3 4 5 6 7 8 9 Strongly agree

Comments to justify response (optional) _____________________________

Suggested changes in terminology or additional medication/medication classes for this category (optional) _____________________

***The following medications and/or medication classes relate to anti-anaemic preparations, blood substitutes and perfusion solutions***

I believe IRON DEXTRAN (PARENTERAL) is a high-risk medication and should be included in a high-risk medication list for use in Australian residential aged care

Strongly disagree 1 2 3 4 5 6 7 8 9 Strongly agree

I believe ALL PARENTERAL NUTRITIONAL PREPARATIONS should be considered high-risk medications and should be included in a high-risk medication list for use in Australian residential aged care

Strongly disagree 1 2 3 4 5 6 7 8 9 Strongly agree

I believe ALL POTASSIUM AND OTHER ELECTROLYTES should be considered high-risk medications and should be included in a high-risk medication list for use in Australian residential aged care

Strongly disagree 1 2 3 4 5 6 7 8 9 Strongly agree

Comments to justify response (optional) _____________________________

Suggested changes in terminology or additional medication/medication classes for this category (optional) _____________________

******* Page 6 *******

**Category 3 – CARDIOVASCULAR SYSTEM**

***The following medications and/or medication classes relate to antihypertensives and beta-blocking agents.***

I believe PROPRANOLOL is a high-risk medication and should be included in a high-risk medication list for use in Australian residential aged care

Strongly disagree 1 2 3 4 5 6 7 8 9 Strongly agree

I believe ALL BETA-BLOCKERS *(e.g. atenolol, bisoprolol, carvedilol, metoprolol, propranolol, sotalol)* should be considered high-risk medications and should be included in a high-risk medication list for use in Australian residential aged care

Strongly disagree 1 2 3 4 5 6 7 8 9 Strongly agree

I believe ALL ANTIHYPERTENSIVES *(e.g. diuretics, ACE inhibitors, calcium channel blockers, angiotensin II antagonists, central antihypertensives, vasodilators, beta blockers, renin inhibitors, and combination drugs)* should be considered high-risk medications and should be included in a high-risk medication list for use in Australian residential aged care

Strongly disagree 1 2 3 4 5 6 7 8 9 Strongly agree

Comments to justify response (optional) _____________________________

Suggested changes in terminology or additional medication/medication classes for this category (optional) _____________________

***The following medications and/or medication classes relate cardiac therapy.***

I believe DIGOXIN is a high-risk medication and should be included in a high-risk medication list for use in Australian residential aged care

Strongly disagree 1 2 3 4 5 6 7 8 9 Strongly agree

I believe EPINEPHRINE/ADRENALINE (PARENTERAL) is a high-risk medication and should be included in a high-risk medication list for use in Australian residential aged care

Strongly disagree 1 2 3 4 5 6 7 8 9 Strongly agree

Comments to justify response (optional) _____________________________

Suggested changes in terminology or additional medication/medication classes for this category (optional) _____________________

***The following medications and/or medication classes relate diuretics.***

I believe EPLERENONE is a high-risk medication and should be included in a high-risk medication list for use in Australian residential aged care

Strongly disagree 1 2 3 4 5 6 7 8 9 Strongly agree

I believe SPIRONOLACTONE is a high-risk medication and should be included in a high-risk medication list for use in Australian residential aged care

Strongly disagree 1 2 3 4 5 6 7 8 9 Strongly agree

I believe ALL **LOOP** DIURETICS *(e.g. furosemide, bumetanide)* should be considered high-risk medications and should be included in a high-risk medication list for use in Australian residential aged care

Strongly disagree 1 2 3 4 5 6 7 8 9 Strongly agree

I believe ALL DIURETICS should be considered high-risk medications and should be included in a high-risk medication list for use in Australian residential aged care

Strongly disagree 1 2 3 4 5 6 7 8 9 Strongly agree

Comments to justify response (optional) _____________________________

Suggested changes in terminology or additional medication/medication classes for this category (optional) _____________________

******* Page 7 *******

**Category 4 – MUSCULO-SKELETAL SYSTEM**

***The following medications and/or medication classes relate to musculoskeletal medications.***

I believe ALL BISPHOSPHONATES *(e.g. risedronate, alendronate, zoledronic acid)* should be considered high-risk medications and should be included in a high-risk medication list for use in Australian residential aged care

Strongly disagree 1 2 3 4 5 6 7 8 9 Strongly agree

I believe DRUGS OTHER THAN BISPHONSPHONATES THAT AFFECT BONE STRUCTURE AND MINERALIZATION *(e.g. denosumab)* should be considered high-risk medications and should be included in a high-risk medication list for use in Australian residential aged care

Strongly disagree 1 2 3 4 5 6 7 8 9 Strongly agree

I believe ALL TOPICAL PRODUCTS FOR JOINT AND MUSCULAR PAIN *(e.g. NSAID gel/patch, capsaicin cream, fentanyl patch)* should be considered high-risk medications and should be included in a high-risk medication list for use in Australian residential aged care

Strongly disagree 1 2 3 4 5 6 7 8 9 Strongly agree

I believe ALL NON-STEROIDAL ANTI-INFLAMMATORY DRUGS (NSAIDS) should be considered high-risk medications and should be included in a high-risk medication list for use in Australian residential aged care

Strongly disagree 1 2 3 4 5 6 7 8 9 Strongly agree

I believe ALL ANTI-INFLAMMATORY MEDICATIONS AND ANTI-RHEUMATIC MEDICATIONS *(e.g. celecoxib, diclofenac, indomethacin, meloxicam, naproxen, hydroxychloroquine)* should be considered high-risk medications and should be included in a high-risk medication list for use in Australian residential aged care

Strongly disagree 1 2 3 4 5 6 7 8 9 Strongly agree

Comments to justify response (optional) _____________________________

Suggested changes in terminology or additional medication/medication classes for this category (optional) _____________________

******* Page 8 *******

**Category 5 – NERVOUS SYSTEM**

***The following medications and/or medication classes relate to analgesics.***

I believe PARACETAMOL (ACETAMINOPHEN) Is a high-risk medication and should be included in a high-risk medication list for use in Australian residential aged care

Strongly disagree 1 2 3 4 5 6 7 8 9 Strongly agree

I believe MORPHINE Is a high-risk medication and should be included in a high-risk medication list for use in Australian residential aged care

Strongly disagree 1 2 3 4 5 6 7 8 9 Strongly agree

I believe METHADONE Is a high-risk medication and should be included in a high-risk medication list for use in Australian residential aged care

Strongly disagree 1 2 3 4 5 6 7 8 9 Strongly agree

I believe CODEINE Is a high-risk medication and should be included in a high-risk medication list for use in Australian residential aged care

Strongly disagree 1 2 3 4 5 6 7 8 9 Strongly agree

I believe OXYCODONE Is a high-risk medication and should be included in a high-risk medication list for use in Australian residential aged care

Strongly disagree 1 2 3 4 5 6 7 8 9 Strongly agree

I believe TRAMADOL Is a high-risk medication and should be included in a high-risk medication list for use in Australian residential aged care

Strongly disagree 1 2 3 4 5 6 7 8 9 Strongly agree

I believe ALL OPIOIDS should be considered high-risk medications and should be included in a high-risk medication list for use in Australian residential aged care

Strongly disagree 1 2 3 4 5 6 7 8 9 Strongly agree

I believe ALL ANALGESICS should be considered high-risk medications and should be included in a high-risk medication list for use in Australian residential aged care

Strongly disagree 1 2 3 4 5 6 7 8 9 Strongly agree

Comments to justify response (optional) _____________________________

Suggested changes in terminology or additional medication/medication classes for this category (optional) _____________________

***The following medications and/or medication classes relate to antiepileptics.***

I believe CARBAMAZEPINE is a high-risk medication and should be included in a high-risk medication list for use in Australian residential aged care

Strongly disagree 1 2 3 4 5 6 7 8 9 Strongly agree

I believe NARROW THERAPEUTIC RANGE ANTIEPILEPTICS *(e.g. phenytoin)* should be considered high-risk medications and should be included in a high-risk medication list for use in Australian residential aged care

Strongly disagree 1 2 3 4 5 6 7 8 9 Strongly agree

I believe ALL ANTIEPILEPTICS should be considered high-risk medications and should be included in a high-risk medication list for use in Australian residential aged care

Strongly disagree 1 2 3 4 5 6 7 8 9 Strongly agree

Comments to justify response (optional) _____________________________

Suggested changes in terminology or additional medication/medication classes for this category (optional) _____________________

***The following medications and/or medication classes relate to anti-Parkinson drugs.***

I believe ALL ANTI-PARKINSON MEDICATIONS should be considered high-risk medications and should be included in a high-risk medication list for use in Australian residential aged care

Strongly disagree 1 2 3 4 5 6 7 8 9 Strongly agree

Comments to justify response (optional) _____________________________

Suggested changes or additional medication/medication classes for this category (optional) _____________________

***The following medications and/or medication classes relate to psycholeptics.***

I believe AMITRIPTYLINE Is a high-risk medication and should be included in a high-risk medication list for use in Australian residential aged care

Strongly disagree 1 2 3 4 5 6 7 8 9 Strongly agree

I believe MIRTAZAPINE Is a high-risk medication and should be included in a high-risk medication list for use in Australian residential aged care

Strongly disagree 1 2 3 4 5 6 7 8 9 Strongly agree

I believe SERTRALINE Is a high-risk medication and should be included in a high-risk medication list for use in Australian residential aged care

Strongly disagree 1 2 3 4 5 6 7 8 9 Strongly agree

I believe ALL ANTIDEPRESSANTS should be considered high-risk medications and should be included in a high-risk medication list for use in Australian residential aged care

Strongly disagree 1 2 3 4 5 6 7 8 9 Strongly agree

I believe LITHIUM is a high-risk medication and should be included in a high-risk medication list for use in Australian residential aged care

Strongly disagree 1 2 3 4 5 6 7 8 9 Strongly agree

I believe HALOPERIDOL is a high-risk medication and should be included in a high-risk medication list for use in Australian residential aged care

Strongly disagree 1 2 3 4 5 6 7 8 9 Strongly agree

I believe QUETIAPINE is a high-risk medication and should be included in a high-risk medication list for use in Australian residential aged care

Strongly disagree 1 2 3 4 5 6 7 8 9 Strongly agree

I believe ALL ANTIPSYCHOTICS should be considered high-risk medications and should be included in a high-risk medication list for use in Australian residential aged care

Strongly disagree 1 2 3 4 5 6 7 8 9 Strongly agree

I believe DIAZEPAM is a high-risk medication and should be included in a high-risk medication list for use in Australian residential aged care

Strongly disagree 1 2 3 4 5 6 7 8 9 Strongly agree

I believe ALL BENZODIAZEPINES should be considered high-risk medications and should be included in a high-risk medication list for use in Australian residential aged care

Strongly disagree 1 2 3 4 5 6 7 8 9 Strongly agree

I believe ALL Z-DRUGS (e.g. zolpidem, zopiclone) should be considered high-risk medications and should be included in a high-risk medication list for use in Australian residential aged care

Strongly disagree 1 2 3 4 5 6 7 8 9 Strongly agree

Comments to justify response (optional) _____________________________

Suggested changes in terminology or additional medication/medication classes for this category (optional) _____________________

******* Page 9 *******

**Category 6 –** **SYSTEMIC HORMONAL PREPARATIONS**

***The following medications and/or medication classes relate to systemic hormonal preparations (excluding sex hormones and insulin which have been covered previously).***

I believe CORTICOSTEROIDS FOR LONG TERM USE are a high-risk medication and should be included in a high-risk medication list for use in Australian residential aged care

Strongly disagree 1 2 3 4 5 6 7 8 9 Strongly agree

I believe CALCITONIN is a high-risk medication and should be included in a high-risk medication list for use in Australian residential aged care

Strongly disagree 1 2 3 4 5 6 7 8 9 Strongly agree

I believe ALL PARATHYROID HORMONES *(e.g. teriparatide)* should be considered high-risk medications and should be included in a high-risk medication list for use in Australian residential aged care

Strongly disagree 1 2 3 4 5 6 7 8 9 Strongly agree

Comments to justify response (optional) _____________________________

Suggested changes in terminology or additional medication/medication classes for this category (optional) _____________________

******* Page 10 *******

**Category 7 - ANTI-INFECTIVES**

***The following medications and/or medication classes relate to anti-infectives for systemic use.***

I believe ALL SYSTEMIC ANTIBIOTICS should be considered high-risk medications and should be included in a high-risk medication list for use in Australian residential aged care

Strongly disagree 1 2 3 4 5 6 7 8 9 Strongly agree

I believe ALL ANTIMICROBIALS should be considered high-risk medications and should be included in a high-risk medication list for use in Australian residential aged care

Strongly disagree 1 2 3 4 5 6 7 8 9 Strongly agree

Comments to justify response (optional) _____________________________

Suggested changes in terminology or additional medication/medication classes for this category (optional) _____________________

******* Page 11 *******

**Category 8 – ANTINEOPLASTIC AND IMMUNOMODULATING AGENTS**

***The following medications and/or medication classes relate to immunosuppressants and antineoplastic agents.***

I believe ALL **ORAL** CYTOSTATICS *(e.g. cyclophosphamide, fluorouracil, methotrexate)*  should be considered high-risk medications and should be included in a high-risk medication list for use in Australian residential aged care

Strongly disagree 1 2 3 4 5 6 7 8 9 Strongly agree

I believe ALL CHEMOTHERAPEUTIC AGENTS should be considered high-risk medications and should be included in a high-risk medication list for use in Australian residential aged care

Strongly disagree 1 2 3 4 5 6 7 8 9 Strongly agree

Comments to justify response (optional) _____________________________

Suggested changes in terminology or additional medication/medication classes for this category (optional) _____________________

***The following medications and/or medication classes relate to immunosuppressants.***

I believe METHOTREXATE (ORAL, NON-ONCOLOGICAL) is a high-risk medication and should be included in a high-risk medication list for use in Australian residential aged care

Strongly disagree 1 2 3 4 5 6 7 8 9 Strongly agree

Comments to justify response (optional) _____________________________

Suggested changes in terminology or additional medication/medication classes for this category (optional) _____________________

***The following medications and/or medication classes relate to endocrine therapy.***

I believe SELECTIVE OESTROGEN RECEPTOR MODULATORS (e.g. tamoxifen, toremifene) should be considered high-risk medications and should be included in a high-risk medication list for use in Australian residential aged care

Strongly disagree 1 2 3 4 5 6 7 8 9 Strongly agree

Comments to justify response (optional) _____________________________

Suggested changes in terminology or additional medication/medication classes for this category (optional) _____________________

******* Page 12 *******

**Please suggest any additional medication or medication classes, which have not been considered previously in this questionnaire, and which should be considered high-risk medications and should be included in a high-risk medication list for use in Australian residential aged care.**

**Click **here** to see a list of medications and medication classes that have been considered**

| **Category 1 – Alimentary Tract and Metabolism** | Insulin  Metformin  All sulphonylureas  All oral hypoglycemic medications  All anti-diabetic agents  Vitamin D and Vitamin D analogues  Calcium |
| --- | --- |
| **Category 2 – Blood and Blood Forming Organs** | Warfarin  All oral anticoagulants  All anticoagulants  All antiplatelets (including aspirin)  Iron dextran (parenteral)  All parenteral nutritional preparations  All potassium and other electrolytes |
| **Category 3 – CARDIOVASCULAR SYSTEM** | Propranolol  All beta-blockers  All antihypertensives  Digoxin  Epinephrine/Adrenaline (parenteral)  Eplerenone  Spironolactone  All loop diuretics  All diuretics |
| **Category 4 – MUSCULO-SKELETAL SYSTEM** | All bisphosphonates  Drugs other than bisphosphonates that affect bone structure and mineralization  All topical products for joint and muscular pain  All NSAIDs  All anti-inflammatory medications and anti-rheumatic medications |
| **Category 5 – NERVOUS SYSTEM** | Paracetamol  Morphine  Methadone  Codeine  Oxycodone  Tramadol  All opioids  All analgesics  Carbamazepine  Narrow therapeutic range antiepileptics  All antiepileptics  All anti-Parkinson medications  Amitriptyline  Mirtazapine  Sertraline  All antidepressants  Lithium  Haloperidol  Quetiapine  All antipsychotics  Diazepam  All benzodiazepines  All z-drugs |
| **Category 6 –** **SYSTEMIC HORMONAL PREPARATIONS** | Corticosteroids for long term use  Calcitonin  All parathyroid hormones |
| **Category 7 - ANTI-INFECTIVES** | All systemic antibiotics  All antimicrobials |
| **Category 8 – ANTINEOPLASTIC AND IMMUNOMODULATING AGENTS** | All oral cytostatics  All chemotherapeutic agents  Methotrexate  Selective oestrogen receptor modulators |

******* Page 13 *******

Thank you for completing the Round 1 survey.

You will receive a link to complete the Round 2 survey in approximately 1-2 weeks.

We appreciate your support of this study. Please don’t hesitate to contact me if you have any questions or concerns.
