# Supplementary material for: Development of a High‐Risk Medication List for Australian Residential Aged Care: A Modified Delphi Study
Source: Australas J Ageing. 2026 Feb 26;45(1):e70141. doi: 10.1111/ajag.70141 (PMC12945874; doi:10.1111/ajag.70141)
Supplement: Supplementary file 3 — File S3: ajag70141‐sup‐0003‐FileS3.docx. [file AJAG-45-0-s002.docx]

Supplementary file: 3 Scoping Review PRISMA

**Identification of studies via other methods**

**Identification of studies via databases and registers**

Records identified from:

Citation searching (n = 1)

Records removed *before screening*:

Duplicate records removed (n = 258)

Records identified from*:

Databases (n = 809)

**Identification**

Records title/abstract screened

(n = 551)

Records excluded

(n = 504)

Reports not retrieved

(n = 0)

Reports sought for retrieval

(n = 1)

Reports sought for retrieval

(n = 47)

Reports not retrieved

(n = 0)

**Screening**

Reports excluded:

Wrong setting (n=6)

Wrong outcome (not-high risk medication) (n=19)

No full text and insufficient details to determine eligibility (n=13)

Reports excluded: (n= 0)

Reports assessed for eligibility

(n = 1)

Reports assessed for eligibility

(n = 47)

Studies included in review

(n = 10)

**Included**

*PRISMA diagram Source: Page MJ, et al. BMJ 2021;372:n71. doi: 10.1136/bmj.n71.
