# Supplementary material for: Development of a High‐Risk Medication List for Australian Residential Aged Care: A Modified Delphi Study
Source: Australas J Ageing. 2026 Feb 26;45(1):e70141. doi: 10.1111/ajag.70141 (PMC12945874; doi:10.1111/ajag.70141)
Supplement: Supplementary file 4 — File S4: ajag70141‐sup‐0004‐FileS4.docx. [file AJAG-45-0-s004.docx]

**Supplementary File 4:** Characteristics of studies identified in scoping review and used to define Round 1 statements for Delphi study

| **First author, year, country** | **Study type, setting and/or participants** | **Definition of high-risk medication from the study** | **List of medications defined as high-risk in the study** |
| --- | --- | --- | --- |
| Anathhanam, 2012, UK^33^ | Narrative review on patient safety in older people (including nursing homes) | Associated with increased risk of harm in older people but still have a place in chronic disease management. | Anticoagulation for atrial fibrillation  Antipsychotics  Opioids |
| Boockvar, 2009, USA^28^ | Medical record review of 208 residents hospitalised from seven nursing homes. | Defined in the Institute for Healthcare Improvement’s High Alert Medication drug list and those in high-risk drug classes for nursing home patients. | Anticoagulants  Antipsychotics  Digoxin  Insulins  NSAIDs  Opioid Analgesics  Sedatives/hypnotics |
| Fauvelle, 2014, France^30^  Fauvelle, 2015, Fance^52^  Fauvelle, 2017, France^53^ | Medical records of 495 residents of eight nursing homes  *(2015 paper is follow-up of 334 residents from 2014 paper; 2017 paper is summary of 2014 & 2015 results)* | Medications considered most at risk of serious iatrogenicity according to the literature and according to monthly accident tracking responsible for hospitalisations in the acute geriatric service at GHI Le Raincy-Montfermeil  (for which they wrote specific detailed surveillance follow-up parameters) | Anticoagulants - antivitamin K, heparins and derivatives,  Oral antidiabetics - metformin, sulfonylureas, glinides  Antihypertensives – diuretics, ACE inhibitors, calcium channel blockers, angiotensin II antagonists, central antihypertensives, vasodilators, beta blockers, renin inhibitors and combination drugs  Psychotropics - antidepressants, neuroleptics, benzodiazepines, hypnotics, anxiolytics |
| Jokanovic, 2019, Australia^27^ | 22 coroner reports of medication-related deaths from RACFs | Medications most frequently implicated in deaths, and most common medication errors occurring at the stage of monitoring that contributed to deaths (e.g. failure to review/recognise signs of toxicity). Anatomical Therapeutic Chemical (ATC) classification and APINCH (Anti-infectives, Potassium and concentrated electrolytes, Insulin, Narcotics and sedatives, Chemotherapy agents and Heparin and other anticoagulants). | Antidepressants - amitriptyline, sertraline, mirtazapine  Antipsychotics - haloperidol, quetiapine, carbamazepine  Anxiolytic - diazepam  Blood glucose lowering agents - Metformin  Cardiovascular agents –Digoxin, propranolol  Lithium  Opioids - morphine, methadone, codeine, oxycodone, tramadol  Paracetamol  Warfarin |
| Kane-Gill, 2021, USA^29^ | 2127 residents from four nursing homes | Defined as per the National Action Plan for Adverse Drug Event Prevention | Anticoagulants  Antidiabetic agents  Antimicrobials  Antiplatelet drugs  Opioids |
| Sanchez Del Moral, 2021, Spain^32^ | 81 residents from one nursing home | High alert medications are those that, when they are not being properly used, are more likely to cause serious or even fatal harm to patients.  Defined as per High alert medications for patients with chronic illnesses (HAMC) list | Oral anticoagulants  Narrow therapeutic range antiepileptics  Antiplatelets (including aspirin)  Antipsychotics  Beta-blockers  Benzodiazepines and analogues  Corticosteroids long term use  Oral cytostatics  Oral hypoglycemics drugs  Immunosuppressants  Insulins  Loop diuretics - eplerenone/spironolactone  NSAIDs  Opioid analgesics |
| Sluggett, 2020, Global^8^ | Overview of prevalence of high-risk medications in LTCFs | A-PINCH and ISMP list of High-Alert Medications in Long-Term Care Settings  Medications associated with significant harm or death if they are misused or used in error. | Antimicrobials  Antipsychotics  Antithrombotics  Chemotherapeutic agents  Digoxin  Epinephrine (parenteral)  Heparin and other anticoagulants  Oral hypoglycemics  Insulin  Iron dextran (parenteral)  Methotrexate (oral, non-oncology use)  Narcotics (opioids) and other sedatives  Parenteral nutritional preparations  Potassium and other electrolytes |
| Taxis, 2017, Australia and Netherlands^31^ | Pharmacy supply data for 26 nursing homes in Australia (1,560 residents) and six nursing homes in Netherlands (2,037 residents) | Medications known to be problematic in older adults | Systemic antibiotics  Osteoporosis medication - Vitamin A and D, Vitamin D and analogues, Calcium, Selective oestrogen receptor modulators, Teriparatide, Parathyroid hormone, Calcitonin, Bisphosphonates, Bisphosphonate combinations and other drugs affecting bone structure and mineralisation  Pain- Anti-inflammatory or Antirheumatic products, topical product for joint and muscular pain, Analgesics  Psychotropic –Antipsychotic, Anxiolytic, Hypnotic, Sedative, Antidepressant |
| *Bell 2022, Australia [Unpublished expert panel]* | *Nominal group technique involving 8 participants* | *Medications associated with significant harm or death if they are misused or used in error.* | *Anticoagulants*  *Antiepileptics/Anticonvulsants*  *Antihypertensives*  *Anti-Parkinson medications*  *Antimicrobials*  *Antineoplastic agents*  *Antiplatelets*  *Antipsychotics*  *Benzodiazepines*  *CNS Depressants*  *Diuretics*  *Oral hypoglycaemics*  *Immunosuppressants*  *Insulin*  *Opioids*  *Rate controlled medications (e.g. digoxin)*  *Z-Drugs* |

**References**

8. Sluggett JK, Harrison SL, Ritchie LA, et al. High-Risk Medication Use in Older Residents of Long-Term Care Facilities: Prevalence, Harms, and Strategies to Mitigate Risks and Enhance Use. Sr Care Pharm. 2020;35(10):419-433.

27. Jokanovic N, Ferrah N, Lovell JJ, et al. A review of coronial investigations into medication-related deaths in residential aged care. Res Social Adm Pharm. 2019;15(4):410-416.

28. Boockvar KS, Liu S, Goldstein N, et al. Prescribing discrepancies likely to cause adverse drug events after patient transfer. Qual Saf Health Care. 2009;18(1):32-36.

29. Kane-Gill SL, Wong A, Culley CM, et al. Transforming the Medication Regimen Review Process Using Telemedicine to Prevent Adverse Events. J Am Geriatr Soc. 2021;69(2):530-538.

30. Fauvelle F, Kabirian F, Bernard A, et al. [Medical treatment of the elderly in residential care: a multicenter cross-sectional study]. Therapie. 2014;69(5):419-426.

31. Taxis K, Kochen S, Wouters H, et al. Cross-national comparison of medication use in Australian and Dutch nursing homes. Age Ageing. 2017;46(2):320-32.

32. Sánchez Del Moral R, García Giménez I, Peláez Bejarano A, et al. 5PSQ-226 Analysis of high alert medication prescriptions in a nursing home. Eur J Hosp Pharm. 2021;28(Suppl 1):A165-A166.

33. Anathhanam S, Powis RA, Cracknell AL, et al. Impact of prescribed medications on patient safety in older people. Ther Adv Drug Saf. 2012;3(4):165-174.

52. Fauvelle F, Kabirian F, Domingues A, et al. [Impact of Geriatric Drug Guidelines on the Quality Requirement of Elderly Patients]. Therapie. 2015;70(6):515-521.

53. Fauvelle F, Kabirian F. Optimising drug prescription and monitoring in elderly nursing home residents: Impact of drug and therapeutics committees. Pharmacien Hospitalier et Clinicien. 2017;52(3):269-275.
