# Supplementary material for: Development of a High‐Risk Medication List for Australian Residential Aged Care: A Modified Delphi Study
Source: Australas J Ageing. 2026 Feb 26;45(1):e70141. doi: 10.1111/ajag.70141 (PMC12945874; doi:10.1111/ajag.70141)
Supplement: Supplementary file 5 — File S5: ajag70141‐sup‐0005‐FileS5.docx. [file AJAG-45-0-s003.docx]

**Supplementary File 5:** High risk medications and medication classes, grouped by ATC anatomical main group, identified in scoping review

|  | Anathhanum 2012 | Boockvar, 2009 | Fauvelle, 2014-17 | Jokanovic, 2019 | Kane-Gill, 2021 | Sanchez Del Moral, 2021 | Sluggett, 2020 | Taxis, 2017 | Bell, 2022* |
| --- | --- | --- | --- | --- | --- | --- | --- | --- | --- |
| ALIMENTARY TRACT AND METABOLISM (A) |  | x | x | x | x | x | x | x | x |
| Drugs used in Diabetes (A10) |  | x | x | x | x | x | x |  | x |
| - Anti-diabetic agents |  |  |  |  | x |  |  |  |  |
| - Glinides |  |  | x |  |  |  |  |  |  |
| - Insulin |  | x |  |  |  | x | x |  | x |
| - Metformin |  |  | x | x |  |  |  |  |  |
| - Oral hypoglycaemics |  |  |  |  |  | x | x |  | x |
| - Sulfonylureas |  |  | x |  |  |  |  |  |  |
| Vitamins (A11) and mineral supplements (A12) |  |  |  |  |  |  | x | x |  |
| - Potassium and other electrolytes |  |  |  |  |  |  | x |  |  |
| - Vitamin A and D |  |  |  |  |  |  |  | x |  |
| - Vitamin D and analogues |  |  |  |  |  |  |  | x |  |
| - Calcium |  |  |  |  |  |  |  | x |  |
| BLOOD AND BLOOD FORMING ORGANS (B) | x | x | x | x | x | x | x |  | x |
| Antithrombotic Agents (B01) | x | x | x | x | x | x | x |  | x |
| - Anticoagulants/Anticoagulants for atrial fibrillation (e.g. antivitamin K, heparins and derivatives) | x | x | x |  | x |  | x |  | x |
| - Warfarin |  |  |  | x |  |  |  |  |  |
| - Oral anticoagulants |  |  |  |  |  | x |  |  |  |
| - Antiplatelets (including aspirin) |  |  |  |  | x | x |  |  | x |
| - Anti-thrombotics |  |  |  |  |  |  | x |  |  |
| Antianemic preparations (B03) |  |  |  |  |  |  | x |  |  |
| - Iron dextran (parenteral) |  |  |  |  |  |  | x |  |  |
| - Parenteral nutritional preparations |  |  |  |  |  |  | x |  |  |
| CARDIOVASCULAR SYSTEM (C) |  | x | x | x |  | x | x |  | x |
| Cardiac therapy (C01) |  | x |  | x |  |  | x |  | x |
| - Digoxin |  | x |  | x |  |  | x |  | x |
| - Epinephrine (parenteral) |  |  |  |  |  |  | x |  |  |
| Diuretics (C03) |  |  |  |  |  | x |  |  |  |
| - Loop diuretics (eplerenone/spironolactone) |  |  |  |  |  | x |  |  |  |
| Beta blocking agents (C07) and other antihypertensives |  |  | x | x |  | x |  |  | x |
| - Beta-blockers |  |  |  |  |  | x |  |  |  |
| - Propranolol |  |  |  | x |  |  |  |  |  |
| - Antihypertensives (diuretics, ACE inhibitors, calcium channel blockers, angiotensin II antagonists, central antihypertensives, vasodilators, beta blockers, renin inhibitors, and combination drugs) |  |  | x |  |  |  |  |  | x |
| SYSTEMIC HORMONAL PREPARATIONS, EXCL SEX HORMONES AND INSULIN (H) |  |  |  |  |  | x |  | x |  |
| Corticosteroids for systemic use (H02) |  |  |  |  |  | x |  |  |  |
| - Corticosteroids long term use |  |  |  |  |  | x |  |  |  |
| Calcium homeostasis (H05) |  |  |  |  |  |  |  | x |  |
| - Teriparatide |  |  |  |  |  |  |  | x |  |
| - Calcitonin |  |  |  |  |  |  |  | x |  |
| - Parathyroid hormone |  |  |  |  |  |  |  | x |  |
| ANTI-INFECTIVES FOR SYSTEMIC USE (J) |  |  |  |  | x |  | x | x | x |
| - Antimicrobials |  |  |  |  | x |  | x |  | x |
| - Systemic antibiotics |  |  |  |  |  |  |  | x |  |
| ANTINEOPLASTIC AND IMMUNOMODULATING AGENTS (L) |  |  |  |  |  | x | x |  | x |
| - Oral cytostatics |  |  |  |  |  | x |  |  |  |
| - Immunosuppressants |  |  |  |  |  | x |  |  | x |
| - Chemotherapeutic agents |  |  |  |  |  |  | x |  |  |
| - Methotrexate (oral, non-oncological use) |  |  |  |  |  |  | x |  |  |
| - Antineoplastic agents |  |  |  |  |  |  |  |  | x |
| - Selective oestrogen receptor modulators |  |  |  |  |  |  |  | x |  |
| MUSCULOSKELETAL SYSTEM (M) |  |  |  |  |  |  |  | x |  |
| Drugs for treatment of bone diseases (MO5) |  |  |  |  |  |  |  | x |  |
| - Bisphosphonates/bisphosphonate combinations and other drugs affecting bone structure and mineralisation |  |  |  |  |  |  |  | x |  |
| NERVOUS SYSTEM (N) | x | x | x | x | x | x | x | x | x |
| *Analgesics (N02)* | *x* | *x* |  | *x* | *x* | *x* | *x* | *x* | *x* |
| - Opioids/narcotics   - Morphine, methadone, codeine, oxycodone, tramadol | x | x |  | x | x | x | x |  | x |
| - NSAIDs |  | x |  |  |  | x |  |  |  |
| - Paracetamol |  |  |  | x |  |  |  |  |  |
| - Anti-inflammatory or anti-rheumatic products |  |  |  |  |  |  |  | x |  |
| - Topical products for joint and muscular pain |  |  |  |  |  |  |  | x |  |
| - Analgesics |  |  |  |  |  |  |  | x |  |
| *Antiepileptics (N03)* |  |  |  |  |  | *x* |  |  | *x* |
| - Narrow therapeutic range antiepileptics |  |  |  |  |  | x |  |  |  |
| - Antiepileptics/anticonvulsants |  |  |  |  |  |  |  |  | x |
| *Anti-Parkinson Drugs (N04)* |  |  |  |  |  |  |  |  | *x* |
| - Anti-Parkinson medications |  |  |  |  |  |  |  |  | x |
| *Psycholeptics (N05)* | *x* | *x* | *x* | *x* |  | *x* | *x* | *x* | *x* |
| - Antipsychotics/neuroleptics   - Haloperidol, quetiapine, carbamazepine | x | x | x | x |  | x | x | x | x |
| - Benzodiazepines |  |  | x |  |  |  |  |  | x |
| - Benzodiazepines and analogues |  |  |  |  |  | x |  |  |  |
| - Sedatives and hypnotics |  | x | x |  |  |  | x | x |  |
| - Anxiolytics   - diazepam |  |  | x | x |  |  |  | x |  |
| - Antidepressants   - amitriptyline, sertraline, mirtazapine |  |  | x | x |  |  |  | x |  |
| - CNS depressants |  |  |  |  |  |  |  |  | x |
| - Z-drugs |  |  |  |  |  |  |  |  | x |
| - Lithium |  |  |  | x |  |  |  |  |  |

x indicates medication/class was mentioned in that study.
*Unpublished expert panel
